# Modulation of subthalamic beta oscillations by movement, dopamine, and deep brain stimulation in Parkinson’s disease

**DOI:** 10.1038/s41531-024-00693-3

**Published:** 2024-04-05

**Authors:** Varvara Mathiopoulou, Roxanne Lofredi, Lucia K. Feldmann, Jeroen Habets, Natasha Darcy, Wolf-Julian Neumann, Katharina Faust, Gerd-Helge Schneider, Andrea A. Kühn

**Affiliations:** 1 Department of Neurology, Charité—Universitätsmedizin Berlin, corporate member of Freie Universität Berlin and Humboldt-Universität zu Berlin, Berlin, Germany; 2 Berlin Institute of Health at Charité Universitätsmedizin Berlin, Berlin, Germany; 3 Department of Neurosurgery, Charité—Universitätsmedizin Berlin, corporate member of Freie Universität Berlin and Humboldt-Universität zu Berlin, Berlin, Germany; 4 Berlin School of Mind and Brain, Charité Universitätsmedizin Medicine, Berlin, Germany; 5 NeuroCure Clinical Research Centre, Charité Universitätsmedizin, Berlin, Germany; 6 DZNE, German Center for Degenerative Diseases, Berlin, Germany

**Keywords:** Parkinson's disease, Biomarkers

## Abstract

Subthalamic beta band activity (13–35 Hz) is known as a real-time correlate of motor symptom severity in Parkinson’s disease (PD) and is currently explored as a feedback signal for closed-loop deep brain stimulation (DBS). Here, we investigate the interaction of movement, dopaminergic medication, and deep brain stimulation on subthalamic beta activity in PD patients implanted with sensing-enabled, implantable pulse generators. We recorded subthalamic activity from seven PD patients at rest and during repetitive movements in four conditions: after withdrawal of dopaminergic medication and DBS, with medication only, with DBS only, and with simultaneous medication and DBS. Medication and DBS showed additive effects in improving motor performance. Distinct effects of each therapy were seen in subthalamic recordings, with medication primarily suppressing low beta activity (13–20 Hz) and DBS being associated with a broad decrease in beta band activity (13–35 Hz). Movement suppressed beta band activity compared to rest. This suppression was most prominent when combining medication with DBS and correlated with motor improvement within patients. We conclude that DBS and medication have distinct effects on subthalamic beta activity during both rest and movement, which might explain their additive clinical effects as well as their difference in side-effect profiles. Importantly, subthalamic beta activity significantly correlated with motor symptoms across all conditions, highlighting its validity as a feedback signal for closed-loop DBS.

## Introduction

Excessive synchronization of subcortical beta band activity (13–35 Hz) recorded from deep brain stimulation (DBS) target structures is a well-established pathophysiological signature for parkinsonian motor symptoms^1^. Along with symptom alleviation, deep brain stimulation (DBS) and dopaminergic medication have been shown to reduce beta power^2–10^. Within the beta range, modulation of lower (13–20 Hz) and higher (20–35 Hz) frequencies have been hypothesized to reflect different pathophysiological mechanisms. Specifically, low beta frequencies (13–20 Hz) have been linked to the hypodopaminergic state^11–14^. In contrast, high beta frequencies (20–35 Hz) are considered correlates of physiological motor circuit activity and decrease in amplitude during movement^15^.

Thus, movement, dopaminergic medication and subthalamic nucleus (STN) DBS have independently been shown to modulate subthalamic beta activity in Parkinson’s disease (PD). However, their distinct or additive effects on beta oscillations in PD have not been studied so far. This is particularly important for the development of closed-loop DBS algorithms that rely on real-time subcortical feedback to regulate DBS^16,17^, because closed-loop algorithms need to account for everyday movements and regular medication intake, in interaction with DBS effects on beta oscillations.

In this study, we aim to characterize the interactions of dopaminergic medication, subthalamic DBS and movement on beta activity of the subthalamic nucleus (STN) of chronically implanted PD patients. We recorded STN local field potentials after chronic DBS from seven patients in four conditions: after withdrawal of dopaminergic medication and DBS, with medication only, with DBS only, and with simultaneous DBS and medication. The patients were recorded during rest and repetitive finger tapping periods. We compared spectral activity and motor performance across all conditions, and unraveled distinct modulating effects of DBS, medication, and movement on subthalamic beta band activity.

## Results

### Medication and DBS reduce symptom severity and improve motor performance

**Fig. 1:**
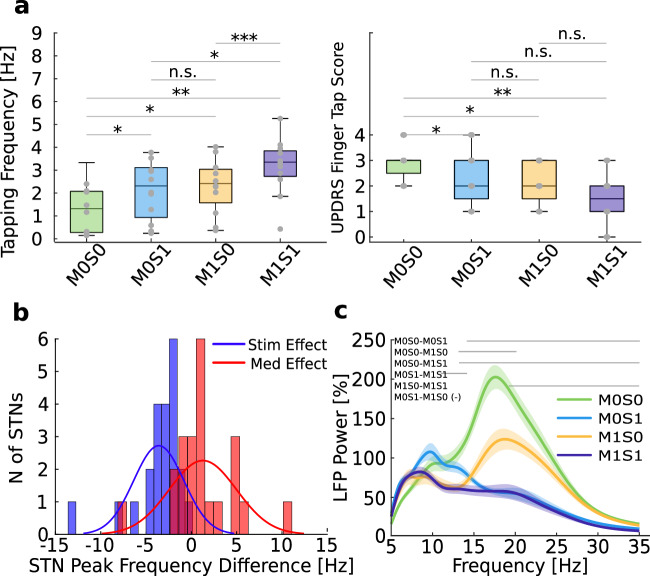
Medication and DBS improve motor performance, reduce symptom severity, and modulate beta band activity during rest. **a** Behavioral metrics (left; tapping frequency, right; Finger tapping score as assessed by UPDRS-Item 3.4) in four different conditions & pair-wise comparisons using a paired-permutation test. The central line on each box indicates the median, while the edges are the 25th/75th percentile. *p < 0.05, **p < 0.01, ***p < 0.001, n.s. not significant. **b** Histograms of the difference in beta peak frequency at rest with DBS (peak frequency in M0S1-M0S0 and M1S1-M1S0 in blue) or with medication (peak frequency in M1S0-M0S0 and M1S1-M0S1 in red). **c** Averaged power spectra of rest activity across recording conditions. Horizontal lines denote statistically significant different clusters between conditions, as resulted from a cluster-based paired permutation test. Shaded areas indicate the standard error of the mean. M0S0 Medication Off-Stimulation Off, M0S1 Medication Off-Stimulation On, M1S0 Medication On-Stimulation Off, M1S1 Medication On-Stimulation On.

To investigate the effect of medication and DBS on motor performance as assessed with a finger tapping task, we compared mean tapping frequencies between all conditions with a paired-permutation test. Compared to the off-medication off-stimulation condition (M0S0), tapping frequency increased similarly with medication (M1S0, paired-permutation, *p* = 0.015) and DBS (M0S1, paired-permutation, *p* = 0.02) with no difference between the two (M1S0 vs M0S1: paired-permutation, *p* = 0.78). When medication and DBS were applied together, tapping frequency was significantly higher than in the medication only (M1S0, paired-permutation, *p* < 0.001) but not the DBS only condition (M0S1, paired-permutation, *p* = 0.12). Similar results were found for clinical ratings of bradykinesia as assessed by UPDRS Item 3.4 (see Table 1 and Fig. 1a).

**Table 1:** Behavioral metrics

Variable	M0S0	M0S1	M1S0	M1S1
Tapping Frequency (Hz)	1.31 ± 1.08^a^	2.13 ± 1.26	2.32 ± 1.17	3.17 ± 1.2
UPDRS 3.4^b^	2.92 ± 0.67	2.25 ± 1.06	2.08 ± 0.79	1.5 ± 1.0

^a^Means ± standard deviations.

^b^UPDRS 3.4 = Unified Parkinson’s disease Rating Scale, Item 3.4.

Other movement metrics, such as the numbers of taps followed a similar pattern, whereas the mean tapping acceleration did not yield significant differences but exhibited similar trends (See Supplementary Table 1).

### Medication and DBS have a differential effect on beta activity at rest

To examine the effect of DBS and medication on beta peak frequencies at rest, we selected the peak frequency between 10–35 Hz in each condition. This was chosen to include spectral widths outside the canonical beta bands. In the absence of medication and DBS (M0S0), beta peaks were detected in all hemispheres of all patients and were always confined to the low beta band (μ = 17.33 ± 2.31 Hz, range = 12–21 Hz). On medication (M1S0) we observed a shift of the beta peak frequency towards the higher beta band while low beta activity was suppressed (μ = 19.33 ± 3.8, paired-permutation, *p* = 0.002). In contrast, DBS (M0S1) was associated with a broader suppression of beta activity including high beta frequencies and a shift of remaining activity towards lower frequencies (μ = 14.92, paired-permutation, *p* = 0.001, Fig. 1b). Individual peak frequencies per condition are provided in Supplementary Table 2.

A cluster-based paired-permutation test revealed differences in beta power across all conditions. In line with previous studies, medication was associated with a decrease in low beta activity (significant cluster: 13–20 Hz) at rest when compared to M0S0 (cluster-based paired-permutation, *p* = 0.03), while DBS was associated with a decrease in broader beta band at rest (significant cluster: 14–35 Hz) when compared to M0S0 (cluster-based paired-permutation*, p* = 0.002). Combined medication and DBS resulted in a broad beta band suppression and was significantly more effective at improving motor performance as compared to medication or DBS alone. Specifically, combined DBS and medication condition led to an additional reduction of higher beta power as compared to medication only (cluster-based paired-permutation, significant cluster: 19–35 Hz, M1S0 vs. M1S1: *p* = 0.005); and vice versa comparing combined stimulation to DBS only condition (M0S1 vs M1S1) led to a trend towards additional suppression of lower beta power (cluster-based paired-permutation, significant cluster: 11–14 Hz, M0S1 vs. M1S1: *p* = 0.06; Fig. 1c). When averaged in the canonical sub-bands of low and high beta, the effect of medication and DBS was similar (see Supplementary Fig. 1, Supplementary Table 3).

### Medication and DBS broaden the beta desynchronization during movement

**Fig. 2:**
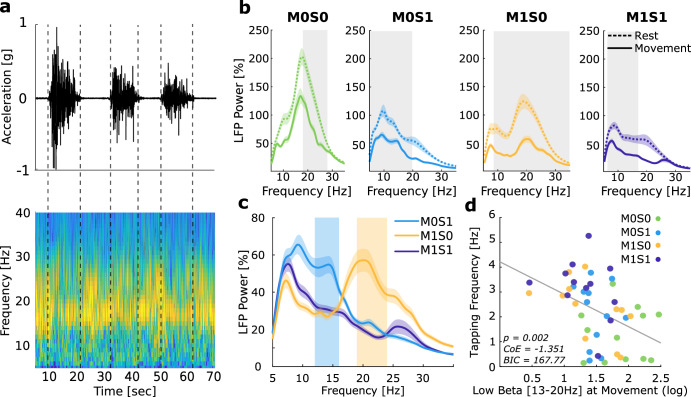
Medication and DBS effects on movement-related beta band activity. **a** Exemplary case of beta desynchronization during movement (Patient #7). Upper plot shows the movement trace of three 10 s finger tapping sequences with 10 s of rest in between. Lower plot shows a time-frequency decomposition of subthalamic oscillations, synchronized with the movement trace. Gray dotted lines indicate the beginning and the end of the movement block. We see a notable suppression of high beta activity (around 20–30 Hz) during the movement periods. **b** Averaged power spectra at rest (dotted lines) and during movement (solid lines) in four different conditions. Shaded gray areas show statistically significant different clusters between rest and movement, as resulted from a cluster-based paired-permutation test. **c** Additive effects of stimulation (yellow line, M1S0) and medication (blue line, M0S1) during movement when compared to the combined state (M1S1, purple line). Significant clusters of medication effect are shown as blue shaded area (M0S1 vs M1S1: 12–16 Hz) and of DBS effect as yellow shaded area (M1S1 vs M1S0: 19–24 Hz). Clusters were identified from a cluster-based paired permutation test. **d** Scatter plot summarizing relation between tapping frequency and low beta band activity during movement across conditions. Gray line indicates the least-square line. Linear Mixed Effects models show that low beta band activity during movement is a strong predictor for motor performance (LME, *p* < 0.002, BIC = 167.96, CoE = −1.305, correlation between predicted and original responses Spearman rho = 0.48, *p* < 0.001).

To investigate the differential modulation of subthalamic beta activity through movement in the presence and absence of medication and DBS, we further analyzed subthalamic beta activity during finger tapping. Movement, in line with previous studies, induced a significant beta band suppression in all conditions (exemplary case shown in Fig. 2a). In the absence of medication and DBS, movement led to a significant suppression between 18–28 Hz (cluster-based paired-permutation*, p* = 0.007). Both medication and DBS broadened the oscillatory frequencies suppressed during movement (cluster-based paired-permutation tests, M1S0: 8–35 Hz, *p* < 0.001; M0S1: 5–20 Hz and 24–35 Hz, *p* = 0.001), see Fig. 2b.

When medication and DBS were combined, we observed an additional suppression of high beta activity as compared to medication alone (cluster-based paired-permutation, M1S0 vs M1S1, identified cluster: 19–24 Hz, *p* = 0.059); and vice versa combined stimulation resulted in an additional suppression of low beta activity as compared to DBS alone (M0S1 vs M1S1) (cluster-based paired-permutation, significant cluster: 12–16 Hz, *p* = 0.045), as shown in Fig. 2c.

These results show that a combination of medication and DBS leads to the largest suppression of beta activity during movement, with medication additionally acting on power of lower beta and DBS on power in higher beta frequencies.

In a sub-analysis we showed that there were no different effects on beta peak, beta power or beta suppression by medication, DBS, and movement, between the patients that were recorded at 3- (*n* = 4 STNs) and 12 months (*n* = 8 STNs) after electrode implantation. Additionally, a similar pattern of beta band modulation was observed for the sub-analysis excluding 3 STNs with different contacts used for Med On - Med Off condition (see Table 2).

**Table 2:** Demographic and clinical information

Patient ID	Age (y)	Sex	DD (y)	Follow Up (months)	LEDD (mg)	UPDRS-III Med Off-Stim Off	UPDRS-III Med Off-Stim On	UPDRS-III Med On-Stim Off	UPDRS-III Med On-Stim On	Stimulation Contact Med Off/On	Recording contact pairs Med Off/On	Contacts selected for chronic stimulation	Stimulation Amplitude Med Off/On (mA)	ECG Artefact
#1	61	f	10	3	775	22	15	5	6	L1/L2	L02/L13	L1	2.0/1.5	Not present
#2	68	m	11	12	1250	54	48	44	33	L2/L2	L13/L13	L1-2 (bipolar)	1.5/ 2.0	Not severe
										R1/R2	R02/R13	R1	2.0/2.5	
#3	63	f	7	3	525	42	24	28	14	L2/L2	L13/L13	L2-1 (bipolar)	2.5/2.0	Severe
										R1/R2	R02/R13	R2-1 (bipolar)	2.0/1.5	
#4	56	f	15	3	150	29	20	11	10	R1/R1	R02/R02	R1	1.5/1.0	Not present
#5	62	f	7	12	0	40	25	28	20	L2/L2	L13/L13	L2	2.0/2.0	Severe
										R2/R2	R13/R13	R2,3 (monopolar)	2.0/2.0	
#6	66	m	16	12	200	50	18	34	16	L2/L2	L13/L13	L2	2.5/2.0	Severe
										R2/R2	R13/R13	R2	2.5/2.5	
#7	56	m	11	12	200	38	8	–	–	L1/L1	L02/L02	L1	2.0/2.0	Not present
										R1/R1	R02/R02	R2	2.0/2.0	

*DD* Disease duration, *LEDD* Levodopa Equivalent Daily Dose.

### Beta activity at rest and movement explains motor performance across medication and DBS conditions

Last, we compared high and low beta power during movement and rest as predictor for motor performance with a linear mixed effects model (LME) across conditions. Here, low beta power at rest was the best predictor of motor performance (LME, *p* < 0.001, BIC = 164.21, CoE = −1.488, correlation between predicted and original responses Spearman rho = 0.52, *p* < 0.001, shown in Supplementary Fig. 2). Low beta power during movement (LME, *p* < 0.002, BIC = 167.96, CoE = −1.305, correlation between predicted and original responses Spearman rho = 0.48, *p* < 0.001, shown in Fig. 2d), as well as high beta during rest and movement were significant predictors of motor performance as well (see Supplementary Fig. 2). This shows that beta activity is a significant predictor of motor performance within patients both at rest and during movement and across stimulation and medication conditions.

## Discussion

In this study, we investigated the interacting effect of dopaminergic medication, DBS and movement on subthalamic beta activity in chronic recordings of PD patients. We showed that medication acts primarily on lower beta frequencies, while DBS provides a stronger suppression of higher beta frequencies. This pattern is similar during movement, although movement-related suppression spread over a larger frequency range during both medication and DBS. Within-patient correlation between subthalamic beta power and tapping frequency across conditions was significant at rest as well as during movement both for low and high beta power. We thus demonstrate that subthalamic beta power is a reliable predictor of motor performance across medication, DBS and movement states.

A previous study by Timmermann et al.^18^ showed that levodopa treatment and DBS had a beneficial effect on tapping movements and pronation-supination. Crucially, they showed that the combination of both treatments is more effective than each therapy alone, making the amplitude and the smoothness of the movement comparable to healthy controls. Here we confirm these findings by showing that motor performance improved when treatment was administered, with an additional effect when both medication and DBS were applied. We further show that medication and DBS have differential effects on subthalamic beta band activity. While medication primarily suppressed low beta activity, the effect of DBS extended to higher beta frequencies. Broad band modulation (8–35 Hz) by medication has been shown before^6^, and stimulation may induce a frequency shift towards lower peak frequencies^4^. Matching this observation, beta peak frequencies varied across conditions, with lower peak frequencies observed more often in the DBS only than the medication only state. These findings suggest that low and high subthalamic beta band might represent distinct networks within the motor circuit, and –accordingly- respond differently to treatment.

The observed distinctive beta suppression patterns of medication and stimulation may translate distinct network effects of both therapies leading to improved motor performance. Previous literature has suggested that low and high beta might be associated with different physiological mechanisms. Enhanced low beta activity has been related to the dopamine-depleted state in PD and is considered a biomarker for bradykinesia and rigidity that drops in parallel with motor improvement^11–14^. This relation is less obvious for the high beta band that is not consistently modulated by dopaminergic medication^13^. Modulation of high beta band was found to be important for successful motor performance^19^ and it has been proposed that high beta might be a physiological inhibitory activity within the hyperdirect pathway^20^. Here, we confirm that medication suppresses predominantly low beta band activity. Importantly, DBS enhances the beta band suppression over a larger frequency range that was associated with better motor performance. This suggests that, even though high beta could be part of a physiological inhibitory mechanism, suppression of it seems to have still an additive therapeutic effect in the context of PD. Future studies may investigate whether persistent suppression of high beta band activity by DBS is associated with known DBS-induced side-effects such as impaired motor inhibition^21,22^.

Successful motor execution has been associated with a desynchronization of subthalamic beta activity^5,7^. Previous studies have shown that patients on medication had larger beta desynchronizations in agreement with improved motor performance^23–25^. However, this effect has mostly been investigated in the acute post-operative period, with externalized electrodes, in the absence of chronic DBS and under influence of a possible post-operative stun-effect. Here, we show movement-related desynchronization of beta band activity in chronically implanted patients in four different treatment states. Our findings confirm that the desynchronization was larger when patients were under dopaminergic medication. Although the relative change during movement with combined medication and DBS is smaller, beta band activity in this condition was significantly reduced, and motor performance was improved in parallel.

Importantly, although beta activity was significantly reduced during movement, it was still a valuable predictor of motor performance when considered across all states. A previous study in a partly overlapping cohort^4^ showed that beta at rest was a significant predictor of velocity in patients across increasing DBS amplitudes. Here, we show that beta during movement correlates with motor performance in all different tested conditions. The combined medication and DBS state is particularly important for future closed-loop DBS algorithms, which aim to be applied in chronically implanted patients that are under both treatments. A very narrow beta band selection used as closed-loop DBS feedback signal may thus be differentially modulated by one of the treatment conditions and a selection of a broader beta band could be useful. Nevertheless, we show that patients under treatment have identifiable beta peaks, and beta activity during movement is still a significant predictor for motor performance.

There are three major limitations to this study. First, our cohort consists of a rather low number of patients. However, the link between subthalamic beta activity and motor impairment in PD has been studied and validated in larger cohorts, some with over 100 PD patients^26^. Additionally, subthalamic changes caused by medication, DBS, and movement have been reported before with a similar number of cases^4,5,11,19,23^. Second, the experimental setting consisted of a short duration of rest and movement, as well as a small number of repetitive movement blocks (three in each condition). Although the duration of rest is relatively short, it has been shown before that 60–100 s of rest is sufficient to provide insights on the effect of DBS and dopaminergic medication^4,7,13^. Similarly, 30–60 s of repetitive movement have been shown to capture movement-related changes^2,18,19,23,25^. The small number of movement blocks did not allow for a trial-based analysis. However, the blocks of repetitive tapping enabled us to rate the motor performance with the UPDRS Item 3.4 for bradykinesia. The experiment was done with the optimal amplitude used to achieve the best clinical effect. The latter was evaluated during a systematic stepwise increase of stimulation amplitude in steps of 0.5 mA until side-effects were reported or up to 3.0 mA. The effects on these stepwise increases on subthalamic beta activity have been reported elsewhere and were not part of this study^4^. Third, beta has been shown to fluctuate in repetitive movements, with a notable desynchronization during motor execution, followed by beta synchronization^25^, something that makes these movement paradigms challenging to disentangle. To tackle these fluctuations, we focused on each tap individually, instead of averaging the whole movement period, thus avoiding the post-tapping beta synchronization.

This study sheds light on the interaction of medication, DBS, and movement, and their distinct effects on subthalamic beta band activity. Crucially, we provide evidence that beta band activity can be reliably recorded in chronically implanted PD patients, and we show distinctive beta suppression patterns (in peak activity, and in amplitude) for medication and DBS, which are similar in both rest and movement states. Moreover, we show that beta during movement is a significant predictor of motor performance. Future more complex paradigms of closed-loop DBS should incorporate therapy-induced changes in a state-dependent manner.

## Methods

### Subjects

All patients gave written informed consent prior to the study. The study protocol was approved by the local ethics committee (EA2/256/60) in accordance with the standards set by the Declaration of Helsinki. All patients with idiopathic PD that were implanted with the Percept and the 3389 electrodes and had an inpatient admission to our clinic in September 2020 – January 2022 for stimulation parameters adjustment were considered for the study. A total of 11 patients with idiopathic PD (six female, mean age = 60.1 ± 6.5 years, mean disease duration = 10.8 ± 3.8 years) that underwent bilateral implantation of DBS-electrodes in the STN participated in our study. Electrode placement was informed by intraoperative microelectrode recordings and confirmed by post-operative imaging. Patients were implanted with the Medtronic (MN, USA) 3389 DBS Leads, which were connected to the sensing-enabled pulse generator (Medtronic Percept).

Patients presenting with a pronounced kinematic tremor that interfered with the movement task (*n* = 3) and patients that did not tolerate withdrawal of medication (*n* = 1) were excluded from analysis, resulting in seven patients included in the final analyses (four female, mean age = 61.7 ± 4.6 years, mean disease duration = 11 ± 3.5 years.) From these seven patients, two did not complete the recording in both hemispheres due to fatigue, resulting in 12 STNs in total. Recordings were performed three months (*n* = 3) or 12 months after pulse generator implantation (*n* = 4). See Table 2 for detailed clinical information.

### Recording protocol

Recordings of subthalamic activity were performed via the sensing-enabled pulse generator in the following four conditions: 1 – medication off / DBS off (referred to as M0S0), 2- medication off / DBS on (referred to as M0S1), 3 – medication on/DBS off (referred to as M1S0), 4- medication on/DBS on (referred to as M1S1). In the off-medication state, patients were withdrawn from dopaminergic medication at least 12 h before the recording. In the on-medication state, patients were administered 100–200 mg (1.5 times their morning dopaminergic medication) of fast acting L-Dopa at least 30 min before the recording. DBS was turned off/on at least 30 min prior to the recording^27^. Because hardware requirements allow only one bipolar recording around the active DBS-contact options (contacts 0–2 and 1–3, with 0 being the lower and 3 the uppermost contact), the contact pair with the most pronounced spectral peak in the beta range (13–35 Hz) was chosen for subthalamic recordings. Accordingly, the middle contact (1 or 2 respectively) was used for DBS. DBS pulse width and frequency were set at 60 µs and 130 Hz across patients. Amplitude was restricted to a maximum of 3 mA and set 0.5 mA below the patient-specific DBS amplitude that was fully tolerated and both rest and movement blocks were performed (μ = 2.0 ± 0.4 mA). Electrode localizations for all patients are presented in Supplementary Fig. 3. DBS was applied unilaterally, starting with the hemisphere contralateral to the most affected body side, resulting in two tested hemispheres per subject.

For each recording condition, the patient was comfortably seated in an armchair and the paradigm consisted of a 60-s-long rest recording, followed by three blocks of 10 s of finger tapping (UPDRS, Item 3.4), with 10 s of rest in between. A tri-axial accelerometer was attached to the distal phalanx of the index finger contralateral to the unilateral DBS.

Each block lasted 2 min. Of note, these blocks were repeated at 0.5 mA steps from the off to the on-stimulation state. These steps were not included in the following analyses but have been reported elsewhere for the off-medication condition^4^. Improvement of overall parkinsonian motor signs was assessed with the MDS UPDRS-III scale. Active DBS-contacts and UPDRS-III scores are summarized in Table 2.

### Data acquisition and analysis

Local field potentials (LFP) from the STN were recorded as a continuous bipolar time series via the BrainSense Streaming setting of the sensing-enabled pulse generator with a sampling frequency of 250 Hz, which provided a full spectrum of 1–125 Hz. BrainSense Streaming was used during all states of on/off DBS to allow comparability among the sessions and was preferred against other brain sensing modalities of the Percept (e.g., BrainSense Survey or Indefinite Streaming) as it allowed of long recordings along with timestamps, enabling the synchronization with the accelerometer data. Accelerometer traces were recorded at 4 kHz, down sampled to 250 Hz to match the subthalamic recordings and high pass filtered at 1 Hz (TMSi Saga). The synchronization of LFP and accelerometer traces was performed offline with the aid of a custom-made synchronization device that produced a visual signal and a vibration. The visual signal of the device, as well as the LFP recording starting time stamp were detected in the videos. The vibration was detectable in the accelerometer traces. With these three –visual signal, vibration, and recording time stamp- we were able to calculate the temporal latency between the starting point of the accelerometer and LFP recording and thus to synchronize the LFP recordings with the movement traces.

#### Movement analysis

The following three behavioral accelerometer-based metrics were calculated: 1 - tapping frequency; 2 – number of taps, and 3 – mean peak acceleration. As a fourth behavioral metric, finger tapping was clinically scored according to 4 – UPDRS item 3.4. These four metrics were calculated for each tapping block and averaged over the three blocks of each condition (Μ0S0, Μ0S1, M1S0, M1S1). Each full tap (opening and closing fingers) follows a double sinusoidal pattern (Supplementary Fig. 4). Positive and negative peaks indicate the point of maximal acceleration when opening and closing the fingers respectively and were detected in a semi-automatic way (function *findpeaks* in Matlab, visual inspection of each peak and manual adjustment when needed). The peaks were counted for each block, resulting in the total number of taps. Tapping frequency (in Hz) was calculated as the total duration of tapping divided by the number of taps. Mean acceleration (in m/s^2^) was the average maximum opening acceleration in each movement block. To investigate LFP activity during movement, maximum opening acceleration peaks in the movement traces in all three blocks were aligned. When averaging over movement time, only periods of tapping were considered, as stopping of movement (when index finger and thumb meet) are associated with overshooting beta activity.

Periods of tapping were defined as mean duration of a full tap (opening and closing fingers until zero-crossing of acceleration before the index finger and the thumb meet), individually for each hand and condition (mean tap duration of 205.4 ± 8.7 ms).

#### Signal processing

Data analysis was performed using MATLAB (The Mathworks, Nattick, Massachussets), and data were further preprocessed and analyzed using the open source PERCEIVE Toolbox^28^ and the Statistical Parametric Mapping Toolbox (SPM12, UCL, London, UK^29^,). Data were high- and low-pass filtered at 5 and 90 Hz respectively to reduce movement and DBS artifacts. A notch filter at 48–52 Hz was applied to remove the artifact of line noise. Last, filtered data were visually inspected for electrocardiogram (ECG) or DBS contamination.

For the data that were contaminated by ECG, a correction was performed using the *Template Subtraction Method*^30^. Briefly, this algorithm identifies the R-peaks in the signal, and QRS epochs are generated by taking 200 ms before and after the detected peaks. The recording is then subtracted from the corresponding epoch in the original LFP signal.

Furthermore, data were transformed to the time-frequency domain using Morlet wavelet with 8 cycles per frequency in steps of 1 Hz, with a sampling rate of 20 Hz and normalized with a sum rescaling to the 53–90 Hz band. This was chosen to allow for comparisons across patients and correct for differences in electrode impedances, which lead to differences in signal-to-noise ratio.

Rest activity was defined as averaged subthalamic activity over 30 s within the 60-s-long rest period in which no movement occurred. For each condition (M0S0, M0S1, M1S0, M1S1), averaged rest and movement activity was extracted separately for the canonical bands of low (13–20 Hz) and high (20–35 Hz) beta band for comparability with previous studies. For comparison of peak frequencies within the broad beta band across investigated conditions, we defined the frequency with the largest amplitude between 10–35 Hz during rest in each condition. This band was selected to include spectral peaks at border frequencies that extended to the canonical (13–35 Hz) beta band range.

#### Statistical analysis

To compare the effect of DBS, medication, and movement on beta band activity, pairwise differences were assessed with a two-sided paired-permutation test. Permutation tests do not require an assumption on the underlying distribution of the data. Briefly, the permutation test randomly shuffles the input data, creates a new distribution, and compares it with the original one to determine how much it deviates. A cluster-based paired-permutation test was used to identify significant clusters of frequencies in the power spectra, because neighboring frequency bins are highly dependent on each other. To investigate the association between beta power and tapping frequency, we used contralateral beta power (either at rest or during movement) as predictor, tapping frequency as response and hemisphere as grouping variable within a linear mixed effects model with a random intercept and a fixed slope. To investigate whether our data were normally distributed, we ran a Lilliefors test. Beta power was not normally distributed and therefore converted into a logarithmic scale as to be included in the linear mixed effects model. Statistical analysis was performed using MATLAB.

### Reporting summary

Further information on research design is available in the Nature Research Reporting Summary linked to this article.

### Supplementary information

Supplementary Material

Reporting Summary

## Data Availability

All analyses were done in Matlab using the open-source Perceive toolbox (https://github.com/neuromodulation/perceive) and the statistical parametric mapping toolbox (SPM12, UCL, London, UK). The data set for this publication is held by the Movement Disorder and Neuromodulation Unit, Department of Neurology, Charité Univesitätsmedizin Berlin, Germany, and access requests can be addressed to Prof. Dr. Andrea A. Kühn, andrea.kuehn@charite.de. Data sharing is restricted to European General Data Privacy Regulation (GDPR) under the health data category, and require lawful definition of data sharing agreements from all data controllers.
