# Supplementary material for: Modulation of subthalamic beta oscillations by movement, dopamine, and deep brain stimulation in Parkinson’s disease
Source: NPJ Parkinsons Dis. 2024 Apr 5;10:77. doi: 10.1038/s41531-024-00693-3 (PMC10997749; doi:10.1038/s41531-024-00693-3)
Supplement: Supplementary file 1 — Supplementary Material [file 41531_2024_693_MOESM1_ESM.pdf]

SUPPLEMENTARY MATERIAL

A. Medication and stimulation effect on number of taps and acceleration

The total number of taps followed a similar pattern to the tapping frequency and the UPDRS-3.4. Fewer taps were executed in the absence of medication and stimulation than in the medication (p = 0.014) or stimulation only state (p = 0.016). When applied separately, medication and stimulation were associated with similar number of taps (p = 0.713). When both medication and stimulation were applied number of taps was significantly higher than in the medication only (p = 0.002) but not in the stimulation only condition (p = 0.101).

Mean peak acceleration was low in the absence of medication and stimulation in comparison to medication and stimulation only state but the differences were not significant (M0S0 vs M1S0: p = 0.077, M0S0 vs M0S1: p = 0.081). When applied separately, medication and stimulation were associated with similar acceleration (p = 0.82). When both medication and stimulation were applied, acceleration was significantly higher than in the medication only (p = 0.039) but not in stimulation only condition (p = 0.129). Means and standard deviations of number of taps and mean peak acceleration are shown in Supplementary table 1.

Supplementary Table 1. Mean (±standard deviation) of total number of taps and mean acceleration

**Variable**	**M0S0**	**M0S1**	**M1S0**	**M1S1**
**Finger Taps (N)**	11.62 ±9.75	19.64 ±11.25	21.36 ±10.38	29.79 ±11.93
**Mean peak acceleration (m/s^2)**	1.59 ±2.21	2.67 ±2.51	2.78 ±1.81	3.54 ±1.68

B. Individual peak frequencies at rest across conditions

Supplementary Table 2. Individual peak frequencies during rest in all conditions.

Patient	STN	Med Off – Stim Off (Hz)	Med Off – Stim On (Hz)	Med On – Stim Off (Hz)	Med On – Stim On (Hz)
**#1**	L	20	18	25	23
**#2**	L	15	13	21	13
	R	16	14	21	17
**#3**	L	18	15	19	14
	R	18	17	19	16
**#4**	R	18	13	16	13
**#5**	L	17	13	19	(-)
	R	18	15	19	13
**#6**	L	12	10	23	10
	R	18	17	10	(-)
**#7**	L	21	21	22	20
	R	17	13	18	14

C. Mean canonical beta bands and comparisons during rest and movement across conditions

During rest low beta power was significantly suppressed in all conditions when compared to the absence of stimulation and medication (M1S0: $p = 0.013$; M0S1: $p = 0.003$; M1S1: $p = 0.005$). There was no difference in low beta power between medication and stimulation conditions (M1S0 vs M0S1, $p = 0.264$; M1S0 vs M1S1, $p = 0.15$; M0S1 vs M1S1, $p = 0.264$). In contrast, averaged high beta power was significantly suppressed only by stimulation ($p = 0.002$) and not medication ($p = 0.197$). When both medication and stimulation were applied, high beta power was similar to the stimulation only condition ($p = 0.477$) but significantly reduced compared to the medication only condition ($p = 0.008$).

Low beta power during movement was significantly suppressed in all conditions when compared to the absence of stimulation and medication (M1S0: $p = 0.03$; M0S1: $p = 0.01$; M1S1: $p = 0.011$). Low beta was significantly suppressed when patients were on medication, and additional stimulation was applied ($p = 0.009$). There was no difference in low beta power during movement between medication and stimulation conditions (M1S0 vs M0S1, $p = 0.535$; M0S1 vs M1S1: $p = 0.127$). In contrast, averaged high beta power during movement was significantly suppressed only by stimulation ($p = 0.002$) and not medication ($p = 0.174$). When both medication and stimulation were applied, high beta power was similar to the stimulation only condition ($p = 0.808$) but lower than in the medication only condition ($p = 0.007$). There was a difference in high beta power during movement between medication and stimulation conditions ($p = 0.007$). Results are shown in Supplementary Figure 1 and summarized in Supplementary Table 3.

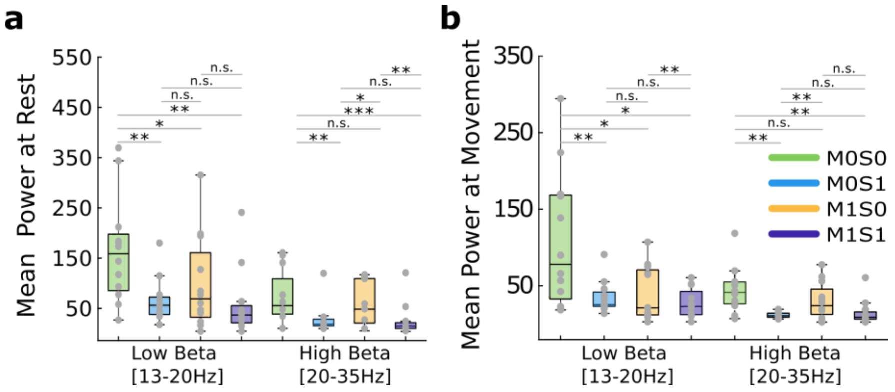

Supplementary Figure 1. Medication and DBS differentially suppress beta band activity with similar effects at rest and movement. Averaged low and high beta band activity in rest (A) and movement (B) across conditions. In both states, medication reduces low beta, while stimulation reduces low and high beta.

Supplementary Table 3. Mean (±standard deviation) power of low (13-20Hz) and high (20-35Hz) in rest and movement.

**Condition**	**Rest**		**Movement**	
	Low Beta	High Beta	Low Beta	High Beta
**M0S0**	165.03 ±105.59	72.21 ±52.22	109.2 ±89.7	44.42 ±29.81
**M0S1**	66.0 ±43.81	28.40 ±29.75	34.77 ±21.13	11.63 ±3.93
**M1S0**	99.2 ±93.56	57.89 ±43.39	40.39 ±35.3	30.25 ±23.88
**M1S1**	58.2 ±67.5	25.46 ±32.71	26.8 ±18.88	14.64 ±16.06

D. Beta band activity during rest and movement is a stable predictor of motor performance across conditions.

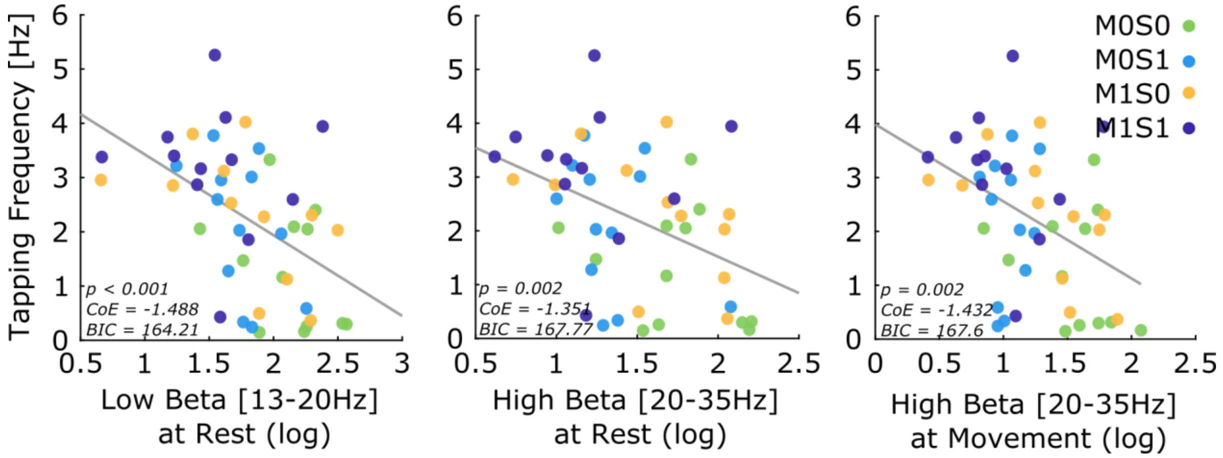

Supplementary Figure 2. Scatter plot summarizing relation between tapping frequency and low beta at rest (left; p < 0.001, BIC = 164.21, CoE = -1.488, correlation between predicted and original responses rho – 0.52, p < 0.001), high beta at rest (middle; p = 0.002, BIC = 167.77, CoE = -1.351, correlation between predicted and original responses rho – 0.42, p = 0.003) and high beta at movement (right; p = 0.002, BIC = 167.6, CoE = -1.432, correlation between predicted and original responses rho – 0.44, p = 0.002) across conditions. Grey line indicates the least-square line.

E. Electrode Localizations

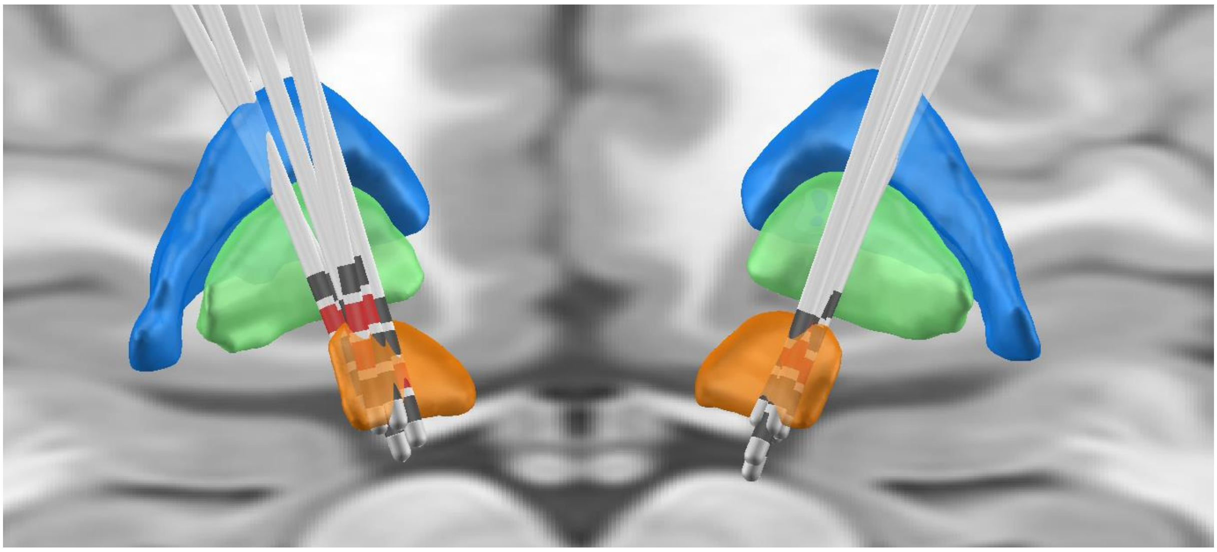

Supplementary Figure 3. DBS lead localization of active DBS contacts. Reconstruction of localization of DBS-leads in the STN (orange). Active DBS contacts used in the current study are marked in red color.

F. Tap detection from movement trace

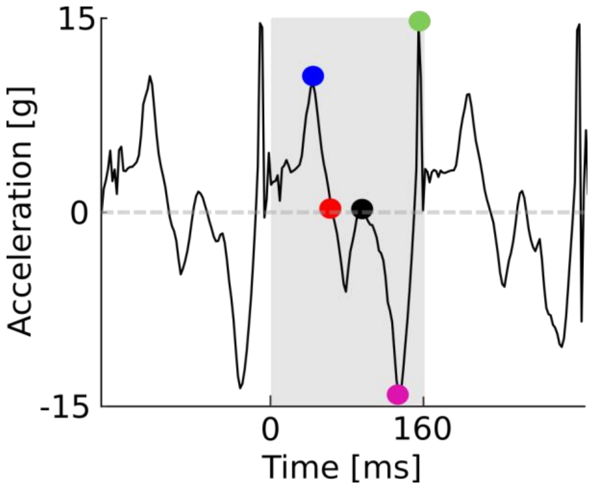

Supplementary Figure 4. Close up of a finger tapping trace (Patient #1). Grey shaded area includes a whole tap. The trace follows a double sinusoidal pattern, in which the positive values of the first sine indicate opening of the index finger and thumb, reaching maximum acceleration at the first positive peak (blue mark) and maximal speed when crossing 0 (red mark). Negative values of the first sine indicate decrease of speed until it reaches 0 (black mark), corresponding to the maximal distance of the fingers. At the

second sine, negative values indicate increased acceleration when closing the fingers until the maximal peak (magenta mark). A spike of acceleration at the end of each tap indicates the ending of the movement, when index finger and thumb meet (green mark).
